# Successional changes of phytodiversity on a short rotation coppice plantation in Oberschwaben, Germany

**DOI:** 10.3389/fpls.2015.00124

**Published:** 2015-03-10

**Authors:** Janine Birmele, Gabriele Kopp, Frank Brodbeck, Werner Konold, Udo H. Sauter

**Affiliations:** ^1^Department of Forest Utilization, Forest Research Institute of Baden-WürttembergFreiburg, Germany; ^2^Institute of Landscape Management, Albert Ludwig University of FreiburgFreiburg, Germany

**Keywords:** short rotation coppice, phytodiversity, *Populus* clones, species richness, Germany

## Abstract

To allow for information on successional changes in phytodiversity over time and space, as well as information on differences between clones and treatments, phytodiversity was monitored on a poplar short rotation coppice plantation in Oberschwaben, Southwest Germany, in four consecutive years. The investigated plantation was divided into two core areas, one planted with poplar clone *Max4*, the other with *Monviso*; each core area was divided into two blocks with alternating treatments: (i) irrigation and fertilization; (ii) irrigation; and (iii) no treatment. All vascular plant species of the ground vegetation were recorded in 72 permanent sampling plots of 25 m^2^ each during vegetation periods using the Braun-Blanquet scale. Results showed that total number of species increased in first 2 years and declined after harvest of the SRC-trees. Total vegetation cover decreased during the 4 years of study. Especially for the two clones there was an opposed trend: grass layer had a high cover on *Monviso* plots, but low cover on *Max4* plots; herb layer the very reverse. However, there was no significant difference between the three treatments compared within each year. Perennial species were dominating over all years, as well as light-demanding species, but their proportion decreased steadily. Our results confirm the conclusion of previous studies which indicate that plant community succession takes place in ground vegetation of SRC and imply that species composition is age-dependent. The selection of clones for SRC can influence ground vegetation; some floristic changes for example caused by different treatments may be visible only when monitored over a longer period of time.

## INTRODUCTION

Biodiversity provides many ecosystem services for example nutrient cycling, water cycling, and pollination. Therefore, it plays an important role in landscape conservation. Due to increasing urbanization and the extension of intensive land use systems, biodiversity has increasingly moved into the focus of research (European Academies Science Advisory Council [EASAC], 2009). It is influenced by the type of land use system and strongly affected by land use changes (Sala et al., 2005). One example for recent land use changes is the establishment of short rotation coppice plantations on former agricultural fields as a result of an increasing demand for woody biomass.

In order to measure and to describe the biodiversity of a given area, animals like ground beetles or birds are often used as indicators (e.g., Sage et al., 2006; Schulz et al., 2009). Since the development of the ground vegetation of a land use system influences the invertebrate communities as well as the food availability and shelter for birds (Dauber et al., 2010), phytodiversity appears to be an appropriate indicator to assess biodiversity (e.g., Weih et al., 2003; Soo et al., 2009). In many of these studies factors influencing phytodiversity were identified by comparing the ground vegetation of plantations of different ages (e.g., Archaux et al., 2010; Baum et al., 2012). One interesting finding is that phytodiversity differs with the plantation age, leading to the conclusion that plant community succession takes place (Delarze and Ciardo, 2002; Britt et al., 2007; Kroiher et al., 2010; Baum et al., 2012). To confirm previous findings on plant community succession (Britt et al., 2007; Kroiher et al., 2010; Baum et al., 2012), it is necessary to observe floristic changes in short rotation coppice plantation over time (Archaux et al., 2010). The current state of knowledge about factors that influence ground vegetation was summarized by Dauber et al. (2010) and Baum et al. (2012). Still of interest is the impact of different species and genotypes used for biomass production (Archaux et al., 2010; Baum et al., 2012).

Therefore in the present study the ground vegetation was monitored on a field scale in four consecutive years and focused on three main questions (see also Cunningham et al., 2004):

(i) Is there a successional trend in ground vegetation?
(ii) Does the type of poplar clone influence phytodiversity in the ground vegetation?
(iii) Has the kind of treatment an influence on phytodiversity?

## MATERIALS AND METHODS

### STUDY AREA

The study area was located in Oberschwaben, Southwestern Germany. It had a slight south-western gradient on an elevation of 630 m above sea level. Mean annual precipitation accounts for 815 mm and mean annual temperature for 7.2^°^C (average 1961–1990, Sigmaringen-Laiz). The soil consists of limestone debris rendzina and terra fuscae. Previously, the site was in agricultural use with a crop rotation of wheat, barley, oat, and ley.

**FIGURE 1:**
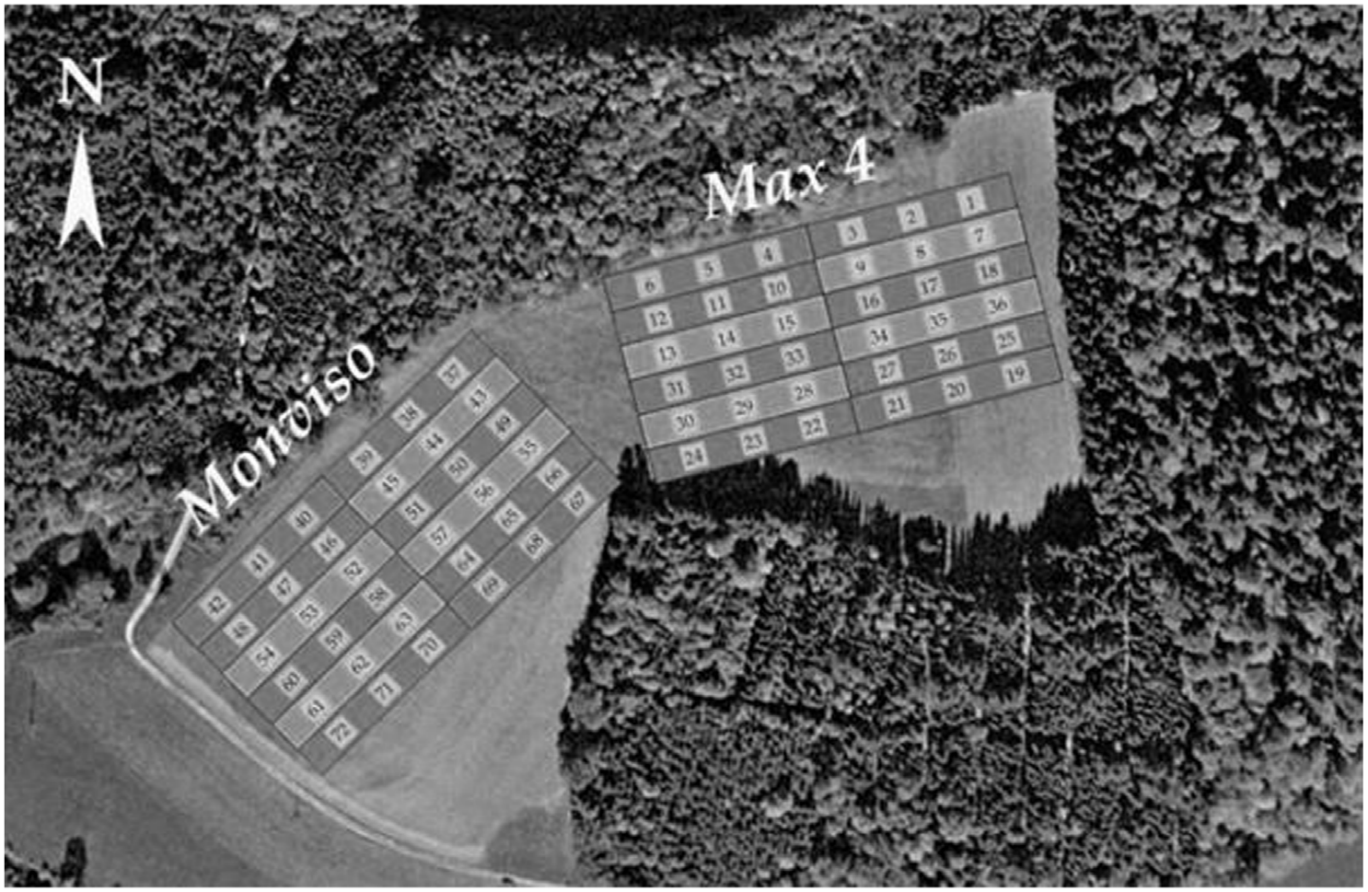
**Study design**. Numbers indicate vegetation sampling plots.

The plantation was established in spring 2009. It has a total size of 4 ha and is divided into two core areas of 1.6 ha each (cf **Figure 1**). Core area 1 was planted with poplar clone *Max4* (*P. nigra × P.maximowiczii*), core area 2 with poplar clone *Monviso* (*P. interamericana × P. nigra*). The plantation is surrounded by mixed forest at three sides (west, north, east) and by meadows in the south.

Both clones were planted in a spacing of 2.5 m by 0.5 m. The core areas were sub-divided into three treatment categories: (i) irrigation (drip irrigation) and fertilization; (ii) irrigation (drip irrigation); and (iii) no treatment as control plots. For this, each area was divided into two blocks with alternating treatments.

Before planting, the area was grubbed, harrowed, and treated with Roundup®. Two days after planting, herbicide was deployed again to repress ground vegetation. The poplars were first harvested in spring 2012. After this, resprouting was suppressed by thistle species *Cirsium arvense,* which thus was combated chemically in June 2012. Poplar pathogen *Melampsora* sp. was first observed on *Monviso* clones in 2011 and was still present during the last sampling in May 2013.

### VEGETATION SAMPLING

Ground vegetation was sampled once during the vegetation periods of each year from 2010 until 2013 (Bräuer, 2010; Birmele, 2013). Sampling plots of 25 m^2^ (5 m × 5 m) were located permanently at a distance of 20 m within poplar rows, and 10 m to adjacent rows. In total, 72 sampling plots were implemented on the study area (36 per clone, 24 per treatment). All vascular plant species of the ground vegetation growing within these plots were compiled in total and subdivided into grass and herb layer. The cover of individual species as well as the cover of grass and herb layer was recorded by using the scale of Braun-Blanquet (1994).

### DATA ANALYSIS

In all study years, species richness and vegetation coverage were recorded. For further description of the development of diversity from 2010 to 2013, as well as to investigate the influence of clone type and treatment on ground vegetation, Shannon-Index and Evenness were calculated (i) for the whole study area, (ii) for *Max4* and *Monviso* core areas, and (iii) for the different treatments. The Shannon-Index H′ (Shannon and Weaver, 1949) and Evenness J′ (Pielou, 1966) were calculated according to following formulas:

$$H\prime =-\Sigma_{i=1}^{R}p_{i}\,\;ln\,\;p_{i}\,\;\;\;\;\;\;\;\;\;\;\;(1)$$

where *p*_i_ is the proportion of individuals belonging to the *i* th species in the dataset.

$$J\prime =\frac{H^{\prime }}{lns}\;\;\;\;\;\;\;(2)$$

where s is the species number.

Besides the development of species richness and vegetation coverage, several floristic attributes were analyzed to characterize the ground flora of the studied short rotation coppice. Ellenberg indicator values for light (L), moisture (F), reaction (R), and nitrogen (N) were used. According to their apparent requirements plants are ranged along a nine point scale; the closer a species’ value to 9, the more it is connected to this indicator. Of all recorded species included in the Ellenberg indicator list the arithmetic mean of Ellenberg indicator values of all plots was calculated. Furthermore, the proportion of species of each year’s species list was calculated for three categories [values 1–3 (species highly connected; e.g., light-demanding); values 4–6 (species moderately connected; e.g., semi-shade plants); values 7–9 (species less connected; e.g., shade tolerant plant)] to illustrate changes in species composition over the 4 years.

To get information on possible successional developments in the species composition, recorded plants were classified as woody species (phanerophytes and nanophanerophytes), perennial species (chamaephytes, hemicryptophytes, cryptophytes, and geophytes) and annual species (therophytes) according to Heilmann et al. (1995). Moreover, recorded species were attached to relevant plant formations, which were derived from Korneck and Sukopp (1988). Vegetation covers with the same physiognomic character are summarized in 24 plant formations. These formations are arranged with increasing complexity of their habit and species composition, they follow a sociological progression.

Statistical analyses were performed using IBM SPSS Statistics 15. Spearman’s rank correlation coefficient was calculated with a significance level of *P* = 0.01.

## RESULTS

### PHYTODIVERSITY ON STUDY AREA

**FIGURE 2:**
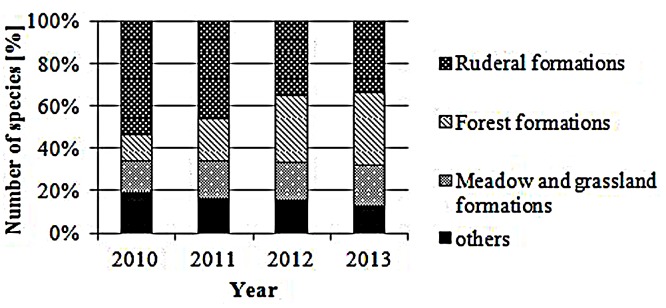
**Plant formations.** Proportion [%] of ruderal, forest, meadow and grassland and other plant species according to Korneck and Sukopp (1988).

In total, 132 different plant species were found in the 72 sample plots within the 4-years study period, most of them associated with ruderal vegetation; in detail, weeds and short-living ruderal vegetation species such as *C. arvense* (Field thistle) and *Myosotis arvensis* (Field forget-me-not) accounted for higher numbers than perennial ruderal herb species and other nitrophilous plant species, such as *Rumex obtusifolius* (Broad-leaved dock) and *Daucus carota* (Wild carrot; cf **Figure 2**). In 2010, subalpine tall forb and shrub vegetation, especially *Arrhenatherum elatius* (False oat grass), accounted for the highest proportion (9.1%). In the other years, species associated with mesophilous fallen leaves forests, for example *Ranunculus repens* (Creeping buttercup), *Rubus idaeus* (Common red raspberry) and *Equisetum arvense* (Field horsetail), as well as very young *Fraxinus excelsior* (Common ash) or *Acer platanoides* (Norway maple) trees, were dominating; their proportion increased from 3.6% in first year to 18.3% in the last year.

Species richness showed a strong incremental trend from 55 species in 2010 to 78 in 2011 and was reduced afterward to 61 species in 2013. The mean number of species for all sampling plots revealed an oppositional trend at first. In 2010, it accounted for 13.4 species, followed by a decrease in 2011, and an increase after harvest (cf **Table 1**). The Shannon-Index resembled the trend of species richness: it increased from 2010 to 2011 and declined in following years, which suggests an increasing number of species or increasing equal distribution in first years and a reduction afterward. SD is highest after harvest, which indicates a large amount of variation. On the other hand, the Evenness indicates that equal distribution decreased over all the 4 years. The decrease was especially noticeable from first to second year. This suggests that the dominance of a few species increased compared to the residual ones. Results of Evenness calculation are supported by the annual percentages of most common species (cf **Table 1**). Especially years 2011, 2012, and 2013 showed similar species occurrences. In 2010, species that appeared in more than 75% of the plots were *D. carota*, *Taraxacum sect. Ruderalia* (Common dandelion), and *Galium aparine* (Cleavers). In the following years, the occurrence of *T. sect. Ruderalia* increased and was accompanied by *Arrhenatherum elatius* which appeared in every single plot until 2013.

**Table 1:** Species richness, mean number of species, Shannon (H_**s**_) and Evenness (**J**′) index and mean cover for whole study area.

Year	Species richness	Mean number of species	Shannon (H_s_)	Evenness (J′)	Mean cover (%)
2010	55	13.4 (3.1)	1.51	0.376	129.0 (16.7)
2011	78	8.5 (3.31)	1.55	0.355	100.3 (25.0)
2012	65	12.3 (4.27)	1.51	0.353	94.1 (17.0)
2013	61	10.2 (3.03)	1.44	0.351	100.2 (27.7)

*SD (s) for mean number of species and mean coverage is shown in brackets. Cover values bigger than 100% result from summarizing herb layer and grass layer which might be overlapping (e.g., grass species growing higher than herb species)*.

Among all recorded ground vegetation species, light-demanding plant species were dominating in all study years. Yet, their proportion showed a steady deterioration from 62 to 39%. At the same time, the proportion of semi-shade species raised from 22 to 34%. In 2012 and 2013, one shade-tolerant species – *G. odoratum* (Woodruff) – occurred. Plot mean Ellenberg light value increased toward the second year, after harvest of the SRC-trees it decreased. Further, species requiring moist sites were dominating considerably over all years with a slightly fluctuating proportion of 49–65%; dry-site indicators accounted for approximately 12% in 2011 and 2012 and had a clearly smaller proportion in the other years. Plot mean Ellenberg moisture value increased in first years and receded slightly in the last year. Species of weakly acid to basic conditions (values 7–9) had the highest share over the study years with a proportion ranging from 46 to 31%. In first 3 years plot Ellenberg reaction showed incrementally higher values than in the last year. Species indicating richly fertile to extremely fertile conditions (values 7–9) were dominating in the first 3 years with 40 to 44%, in 2013 proportion shifts toward species representing intermediate fertility (37%). Plot mean Ellenberg nitrogen value gained higher values toward the second year, after harvest it decreased. Standard deviations are generally similar (cf **Table 2**).

**Table 2:** Plot mean Ellenberg value.

Year	Light (L)			Moisture (F)			Nitrogen (N)			Reaction (R)		
	Total area	*Max4*	*Monviso*	Total area	T1^1^	T2^2^	T3^3^	Total area	T1^1^	T2^2^	T3^3^	Total area
2010	7.04 (0.2)	7.03 (0.97)	7.05 (1.09)	4.78 (0.25)	4.75 (0.35)	4.79 (0.38)	4.78 (0.28)	6.54 (0.27)	6.38 (0.30)	6.64 (0.29)	6.60 (0.16)	6.85 (0.19)
2011	7.13 (0.26)	7.14 (0.8)	7.11 (0.8)	4.86 (0.4)	4.82 (0.58)	4.89 (0.59)	4.84 (0.44)	6.95 (2.19)	6.88 (0.28)	7.01 (0.36)	6.96 (0.37)	6.88 (0.35)
2012	6.85 (0.24)	6.82 (1.1)	6.88 (1.13)	5.11 (0.25)	5.11 (0.29)	5.11 (0.31)	5.12 (0.32)	6.65 (0.29)	6.67 (0.38)	6.68 (0.31)	6.60 (0.37)	7.04 (0.16)
2013	6.62 (0.32)	6.52 (1.3)	6.65 (1.1)	5.08 (0.3)	5.04 (0.43)	5.11 (0.35)	5.07 (0.41)	6.80 (0.38)	6.77 (0.33)	6.77 (0.30)	6.86 (0.24)	6.77 (0.27)

*SD (s) is shown in brackets*.

*^1^Treatment 1 (non-treated control plots); ^2^Treatment 2 (irrigated plots); ^3^Treatment 3 (irrigated and fertilized plots)*.

The majority of species were perennial with their proportion fluctuating in a range between 47 and 52% over the 4 years. Annual species declined from 36% in 2010 to 21%in 2013. Woody species were recorded from 2011 onward with an increasing proportion of 4 to 16%. One invasive neophyte species occurred in year 2013 – *Impatiens parviflora* (Small balsam) –, in one plot with a cover of 62.5%.

The total percentage cover of ground vegetation decreased during the 4 years of study, varying considerably from 40 to 180% on individual plots (values above 100% resulting from summarizing herb layer and grass layer which might be overlapping; cf **Table 1**). Nevertheless mean cover per plot was very high in each year, accounting for approximately 100%.

A positive edge effect was recorded for species richness, indicated by a decreasing number of species with increasing distance from the surrounding forest for years 2010, 2011, and 2012. In 2013, number of species increased with increasing distance to the forest. However, the correlation was only weak (*R^2^* ranging between 0.001 and 0.027).

According to the dispersal types attributed to the recorded species, endozoochory is the most common one. Here, only dispersal by birds and small mammals is possible, since the plantation is fenced in to keep out bigger mammals. Endozoochory is mainly attributed to species bearing palatable fruits like *Rubus idaeus* and *Fragaria vesca* (Wild strawberry). Dispersal by wind (anemochory) has the second highest proportion; anemochory is specifically high for woody species such as *Acer platanoides* and *Tilia cordata* (Small-leaved lime) which were also recorded in ground vegetation cover. Third important dispersal type is dysochory, again a dispersal type related to animals such as birds and small mammals. This type is for example important for *Rubus idaeus*, *Fragaria vesca,* and *Rumex crispus* (Curled dock). So, dispersal by wind, birds, and small mammals are essential for bringing seeds from the surrounding into the plantation.

### PHYTODIVERSITY PER CLONE

On the study area, there were 36 adjoined plots planted with *Max4* or *Monviso*, respectively, on comparable sites. Species richness for *Max4* plots stayed almost constant over the 4 years of study, whereas the index increased considerably on *Monviso* plots from 2010 to 2011 (cf **Table 3**). In the following years, only minor changes in species richness were noticed. Mean numbers of ground vegetation species growing below both clones followed the same tendency over time. However, the increase in mean number of species growing below *Max4* after harvest was considerably higher than below *Monviso*.

**Table 3:** Species richness, mean number of species, Shannon (H_**s**_ ) and Evenness (**J**′) index, and herb and grass layer cover in % grouped for *Max4* and *Monviso* plots.

Year	Species richness		Mean number of species		Shannon (H_s_)		Evenness (J’)		Grass layer cover [%]		Herb layer cover [%]	
	*Max4*	*Monviso*	*Max4*	*Monviso*	*Max4*	*Monviso*	*Max4*	*Monviso*	*Max4*	*Monviso*	*Max4*	*Monviso*
2010	51	42	13.6 (2.87)	13.3 (3.33)	1.474	1.466	0.375	0.392	40.69 (33.23)	64.72 (34.06)	77.5 (18.61)	31.53 (29.39)
2011	50	53	8.7 (2.63)	8.2 (3.90)	1.485	1.476	0.380	0.372	33.33 (25.66)	67.64 (29.51)	64.16 (22.69)	30.0 (26.27)
2012	50	50	15.2 (2.47)	9.4 (10.54)	1.476	1.432	0.377	0.366	33.34 (21.74)	65.59 (29.17)	55.07 (21.33)	40.84 (31.63)
2013	51	49	11.3 (3.09)	9.2 (2.64)	1.369	1.367	0.348	0.351	29.44 (23.54)	59.58 (25.78)	51.38 (25.37)	39.86 (24.04)

*Cover values bigger than 100% result from summarizing herb layer and grass layer which might be overlapping (e.g., grass species growing higher than herb species)*.

Shannon-Index showed higher values on *Max4* plots but followed the same trend below both clones: an incremental trend from 2010 to 2011, which was followed by a decline. This decline was more distinct on *Monviso* plots. There, Evenness decreased from 2010 to 2013, whereas the index on *Max4* plots increased from 2010 to 2011, and then peaked off.

Comparing the total ground vegetation cover on *Max4* and *Monviso* plots within each year, there is a statistical difference between clones in 2010 (*P* = -0.4; *p* < 0.01), 2012 (*P* = 0.3; *p* < 0.01), and 2013 (*P* = 0.6; *p* < 0.01). Vegetation cover on *Max4* plots decreased from 118% in 2010 to 81% in 2013 whereas cover on *Monviso* plots stayed more or less constant.

Splitting up data into herb and grass layer species, grass layer species had a high cover on *Monviso* plots, but only a low cover on *Max4* plots (cf **Table 3**). The herb layer species showed a reverse trend. Significances for herb layer species grown under the two different clones reached *P* = 0.47 (*p* < 0.01), for grass layer species *P* = -0.45 (*p* < 0.01). On *Max4* plots, a steady reduction of both layer covers was recorded. On *Monviso* plots, the grass layer cover raised from 2010 to 2011 whereas the herb cover decreased slightly. After harvest in 2012, the trend for both layers grass layer declined to 65% and herb layer increased remarkably to 41%. In the last year, cover of both layers dropped slightly.

The plot mean Ellenberg light value followed the same trend for both clones, i.e., a slight increment from 2010 to 2011, and a decrease in the following years.

### PHYTODIVERSITY PER TREATMENT

For all treatment types, species richness increased from first to second year (cf **Table 4**). This increasing tendency retained for irrigated plots also in the third year, whereas species richness on irrigated and fertilized plots stayed constant and showed a decrement on control plots. So, before harvest, irrigated plots featured the lowest values, after harvest especially plots without treatment declined sharply, whereas irrigated and irrigated and fertilized plots were almost equal.

**Table 4:** Species richness, mean number of species, Shannon (H_**s**_) and Evenness (J′) index grouped for treatments.

Year	Species richness			Mean number of species			Shannon (H_s_)			Evenness (J′)		
	T1^1^	T2^2^	T3^3^	T1^1^	T2^2^	T3^3^	T1^1^	T2^2^	T3^3^	T1^1^	T2^2^	T3^3^
2010	45	42	46	14.1 (3.40)	13.4 (2.62)	12.8 (3.23)	1.505	1.446	1.498	0.395	0.387	0.391
2011	46	45	50	8.7 (3.16)	8.3 (3.61)	8.4 (3.30)	0.771	1.459	1.492	0.201	0.383	0.381
2012	41	51	50	11.9 (4.15)	12.5 (4.58)	12.4 (4.11)	1.440	1.503	1.498	0.388	0.382	0.383
2013	38	44	45	9.8 (2.06)	10.0 (3.02)	10.8 (3.81)	1.370	1.411	1.449	0.377	0.373	0.381

*^1^Treatment 1 (non-treated control plots); ^2^Treatment 2 (irrigated plots); ^3^Treatment 3 (irrigated and fertilized plots)*.

However, mean number of species had the same development for all treatments: a decline from 2010 to 2011, then a raise to 2012 and again a reduction to 2013. After harvest, there was the biggest variation in mean number of species, especially for irrigated plots. A significant difference could be seen between plots with no treatment in the 4 years (*P* = 0.56, *p* < 0.01), as well as irrigated plots (*P* = 0.45, *p* < 0.05). However, within each year there was no statistical significance between the three treatments.

Shannon-Index was different to previous considerations. Especially for control plots without treatment, diversity was extremely low in year 2011 (*H*_s_ = 0.771). Also, Evenness for control plots was comparatively low in this year (J′ (2011) = 0.201).

Total ground vegetation cover declined for all three treatments about 20% from 2010 to 2013. Comparing the treatments, variation in cover was quite similar in years 2011 and 2013, whereupon irrigated and fertilized plots show a clearly higher range than the other two treatments in 2010 and 2012. Range was very small for irrigated plots in all years. There was a statistical difference between treatments in 2011 (*P* = 0.3; *p* < 0.01) and 2013 (*P* = -0.3; *p* < 0.01).

Analyzing plot mean Ellenberg moisture values, there was no distinct deviation between the three treatments discovered. The same result was found for plot mean Ellenberg nitrogen value (cf **Table 2**).

## DISCUSSION

### SUCCESSIONAL PATTERNS IN THE FLORA

Like Delarze and Ciardo (2002) and Cunningham et al. (2004) we found an increase in species richness and Shannon-Index until the second year after establishment and a decline afterward. This conforms to several other studies which related the decrease of species richness with plantation age to the reduction of light availability (Archaux et al., 2010; Baum et al., 2012). Hence, after harvest of the poplars an increment in species richness would be expectable. Schmidt and Glaser (2009) mentioned that even maximum values of species number can be achieved after harvest. Interestingly, in this study the event of harvesting seemed to have no influence on the trend of species richness of the total area but a strong influence on mean species richness per plot. It is assumed that the bare ground of the recently harvested site was providing a lower barrier to plant spreading within the plantation. Nevertheless, colonization from the surrounding areas was marginal.

Regarding the edge effect on species richness, several studies stated that there is a decrease of mean species richness per plot with increasing distance to forest (Weih et al., 2003; Augustson et al., 2006). This study confirms this trend for the first 3 years after establishment, whereas in the fourth year the effect got lost. Cunningham et al. (2004) attained a similar result in recently planted plantations, but all with an area of more than 5 ha whereas our plantation was only 4 ha in size. They observed a declining species number with distance to the boundary only in the first 2 years of study. Rowe et al. (2011) found no influence on species richness or diversity by distance to the edge at all, observing three willow plantations between 5 and 9 ha, older than 5 years. According to this, it is likely that an edge effect occurs in younger plantations but not necessarily in older ones.

Evenness was reduced over all 4 years which means that the proportion of few species raised compared to the residual ones. These observations, combined with the decreasing species richness, lead to the conclusion that dominant species cause a displacement of inferior ones.

Observed successional patterns like a declining proportion of light-demanding after the second year (for example Delarze and Ciardo, 2002; Kroiher et al., 2010) and a general decrement of annual species (Heilmann et al., 1995; Delarze and Ciardo, 2002) as well as a shift in species composition toward woodland species (Delarze and Ciardo, 2002; Bielefeldt et al., 2008; Archaux et al., 2010; Kroiher et al., 2010) can be supported by our findings. But, contradicting to most studies stating annual species dominating at early stages (Delarze and Ciardo, 2002; Cunningham et al., 2004) we observed the dominance of perennials in all 4 years after establishment. Further, a reduced ground vegetation cover surveyed in this study is not in accordance with Cunningham et al. (2004) and Baum et al. (2012) who recorded an increase of ground vegetation cover in first years after plantation establishment. However, Gustafsson (1987) suggested, that in stands older than 3 years and in longer rotation times, the vegetation cover will be reduced.

These results confirm the conclusion of previous studies which state that plant community succession takes place in ground vegetation of SRC plantations and imply that species composition is age-dependent. Presumably, a specific ground vegetation is going to adjust sometime.

### INFLUENCE OF CLONE TYPE

Our findings indicate differences between ground vegetation composition below the two clones, since *Max4* plots show a high herb layer and low grass layer cover and *Monviso* plots the very reverse (cf **Table 3**). Studies argue that different SRC species and their clones create different conditions for ground vegetation in some way (Heilmann et al., 1995; Bielefeldt et al., 2008; Archaux et al., 2010; Kroiher et al., 2010). Key factor for that seems to be the incidence of light below these poplars which is mainly influenced by leaf shape and rapidness of canopy closure (Bielefeldt et al., 2008). In early stage, *Monviso* was the higher performing clone, both in annual dry matter increment and height; in 2011, it was overtaken by *Max4,* which might be due to an expansion of poplar pathogen *Melampsora* sp. (Poplar leaf rust) to which *Poplar interamericana* clones like *Monviso* are highly sensitive to. It causes a premature defoliation of the poplar trees in early summer which results in an increased availability of light and soil nutrients at the time when ground vegetation is still growing (Archaux et al., 2010). To this, Heilmann et al. (1995) also state that beneath the clone with low canopy closure there is a high cover of specific plant species, which would be *Max4* at the beginning of this study and *Monviso* in later years. However, the spreading of this pathogen was never scientifically measured and mean plot Ellenberg light values do not really reflect this development.

### INFLUENCE OF TREATMENT

Herbicides are claimed to selectively reduce the vegetation cover and being particularly harmful to certain types of species, for example annuals with seeds in the seed bank (Gustafsson, 1987). So, application of herbicides directly after planting and after harvest might be the reason for low numbers of annual species.

According to previous studies, fertilizers creating higher nutrient concentrations in the topsoil significantly reduced species number (Gustafsson, 1987; Soo et al., 2009). In this study, no significant influence of fertilization could be seen, which was stated in (Heilmann et al., 1995) as well. Instead, irrigation seemed to have an impact on species richness. Compared to the control plots the higher water availability on irrigated as well as irrigated and fertilized plots caused an increase in species number. Beside species richness, also the Shannon-index indicated a higher diversity in ground vegetation cover on treated parts of the plantation. However, in this study irrigation and fertilization was not areal, but took place as drip irrigation with liquid fertilizer in one treatment. Within one sampling plot, there were two irrigation lines directly beneath the poplars. It is questionable how much additional water (and fertilizer) for ground vegetation really was available.

Against expectations, the implication of water and fertilizer did not lead to an increase of plot mean Ellenberg moisture and nitrogen values.

However, it has to be considered that this study only had the possibility to analyze phytodiversity during the first 4 years after establishment of the plantation. It is very likely that several floristic changes are only visible in the long run. Therefore it is necessary to monitor ground vegetation of short rotation coppice plantations over a longer period of time, especially to get a more profound knowledge about the influence of different treatments on phytodiversity.

## Conflict of Interest Statement

The authors declare that the research was conducted in the absence of any commercial or financial relationships that could be construed as a potential conflict of interest.
